# Perceptual Dominance in Brief Presentations of Mixed Images: Human Perception vs. Deep Neural Networks

**DOI:** 10.3389/fncom.2018.00057

**Published:** 2018-07-24

**Authors:** Liron Z. Gruber, Aia Haruvi, Ronen Basri, Michal Irani

**Affiliations:** ^1^Department of Neurobiology, Weizmann Institute of Science, Rehovot, Israel; ^2^Department of Computer Science and Applied Mathematics, Weizmann Institute of Science, Rehovot, Israel

**Keywords:** deep neural networks, object recognition, visual perception, vision, visual competition

## Abstract

Visual perception involves continuously choosing the most prominent inputs while suppressing others. Neuroscientists induce visual competitions in various ways to study why and how the brain makes choices of what to perceive. Recently deep neural networks (DNNs) have been used as models of the ventral stream of the visual system, due to similarities in both accuracy and hierarchy of feature representation. In this study we created non-dynamic visual competitions for humans by briefly presenting mixtures of two images. We then tested feed-forward DNNs with similar mixtures and examined their behavior. We found that both humans and DNNs tend to perceive only one image when presented with a mixture of two. We revealed image parameters which predict this perceptual dominance and compared their predictability for the two visual systems. Our findings can be used to both improve DNNs as models, as well as potentially improve their performance by imitating biological behaviors.

## 1. Introduction

These days, the leading algorithms for many computer vision tasks, and also for modeling the visual system specifically, are Deep Neural Networks (DNNs). DNNs are a class of computer learning algorithms that have become widely used in recent years (Lecun et al., 2015). Interestingly, some current DNNs demonstrate a surprising degree of generalization to a variety of other visual tasks (Hue et al., 2016). DNNs that are trained for image recognition (Russakovsky et al., 2015) are found to be useful also in solving totally different visual tasks (Yosinski et al., 2014). These general-purpose algorithms are suggested to be computationally similar to biological visual systems, even more so than less biologically plausible simulations (Kriegeskorte, 2015; Yamins and Dicarlo, 2016).

Moreover, image representation may be similar in trained DNNs and in biological visual systems. A recent study found that humans and DNNs largely agree on the relative difficulties of variations of images (Kheradpisheh et al., 2016). Researchers also found that when the same image is processed by DNNs and by humans or monkeys, the DNN computation stages are strong predictors of human fMRI, MEG, and monkey electrophysiology data collected from visual areas (Cadieu et al., 2014; Khaligh et al., 2014; Yamins et al., 2014; Güçlü and van Gerven, 2015; Cichy et al., 2017; Seeliger et al., 2017). A different study showed that the final DNN computation stage is even a strong predictor of human-perceived shape discrimination (Kubilius et al., 2016). These studies also showed that the more accurate a DNN model is, the stronger its predictive power, challenging vision researchers to create more accurate models based on biological studies of vision.

Alongside with these similarities, the gap between DNNs visual processing and the biological one is still significant. Marking differences like robustness to manipulations (Geirhos et al., 2017), causes of errors (Nguyen et al., 2015), etc. is of great importance to this field (Moosavi-Dezfooli et al., 2017). Exploring these differences by studying known visual phenomena in DNNs, enables both improving current models as well as studying the possible computational nature of the visual system (Rajalingham et al., 2018). Informative phenomena usually involve some kind of challenge to the visual system—multi-stability, illusions, partial informative images, etc. An example of a human visual phenomenon that was studied using computer vision algorithms, is the existence of Minimal Recognizable Configurations (MIRCS) for the human visual system (Ullman et al., 2016). The differences in recognition rates and behavior between humans and the DNNs tested, shed light on the possible nature of this phenomenon. DNNs were also used to explain the emergence of lightness illusions (Corney and Lotto, 2007), which suggest general conclusions about perception's computational nature. Another illusion that emerged from DNN training is the Muller-Lyer geometrical illusion of size (Zeman et al., 2013).

Other perceptual phenomena that can be studied using DNNs are “visual competition” phenomena, where a few competing percepts are potentially perceived. Most visual competition phenomena are dynamic and involve fluctuation in perception throughout time. They are usually referred to as “multi-stable perception.” They are different from our task (detailed below) and more complex to model, as the main challenge is describing the fluctuations causes and dynamics. When perceptual grouping, for example, is not unique (as in the interpretation of Necker cube), a specifically designed DNN model can be used to describe the computation behind the changes in perception throughout time (Kudo et al., 1999). A well-studied dynamic visual competition phenomenon is binocular rivalry. It occurs when dissimilar monocular stimuli are presented to the two eyes. Rather than perceiving a stable, single mixture of the two stimuli, one experiences alternations in perceptual awareness over time (Blake and Tong, 2008). The neuronal source for these visual competition dynamics is still debatable, researches have revealed evidence in both early visual processing and in higher stages along the ventral stream (Logothetis et al., 1996; Logothetis, 1998; Polonsky et al., 2000; Blake and Logothetis, 2002; Tong et al., 2006; Wilson, 2003)

A biological plausible model for the duration of perceptual alterations was offered in (Laing and Chow, 2002), and studies have shown that the cause for the dynamic switching could be both adaptation and noise-driven (Shpiro et al., 2009). Noise-driven time alterations were further modeled using attractor models (Moreno-Bote et al., 2007). Another dynamic multi-stable phenomenon is monocular rivalry, which differ from the binocular one in that the same image is now presented to both eyes. This time it is a superimposed image, and the clarity of the images it is made from fluctuates alternately in time (O'Shea et al., 2017). Another study showed that bi-stable perception is a form of Bayesian sampling, it further demonstrated that using a neural network, one can capture several aspects of experimental data (Moreno-Bote et al., 2011). Whether the processes or computational basis under binocular and monocular rivalry are similar and how they differ is still studied to these days (O'Shea et al., 2009). In this study, as our task did not involve time, we are merely interested in studying the causes of the perceptual dominance occurring already in brief exposures to superimposed images.

Following this, different image parameters had been shown to affect these competing percepts of multi-stable phenomenon. Motion of objects, contrast, luminance, etc. influence these perceptual alternations (Logothetis et al., 1996). Low-level effects were also shown in masking, where a target image is followed by or mixed with a mask (Alam et al., 2014). Practical models predicting detectability were suggested based on the biological visual system (Bradley et al., 2014) and even further tuned to natural image constrains (Schütt and Wichmann, 2017).

In this study, we propose a different visual competition task by briefly presenting mixed images to both humans and pre-trained object recognition DNNs. Similar mixed images were used to study the effects of attention manipulations in a pre-trained DNN (Lindsay, 2015; Lindsay and Miller, 2017). The model was re-trained as a binary classifier and manipulated at different layers to test performance changes. We created a non-dynamic visual competition that enables a comparison with common recognition DNNs, without manipulating their architecture or their training. By mixing two target images we introduced a similar challenge for both the DNN (trained on regular images) and humans (briefly presented with the mixtures). Brief presentations are ideal for investigating early stages of perceptual competition (Carter and Cavanagh, 2007), and eliminates effects of time that are generally not comparable with most DNNs. Inspired by visual competitions researches, we generated a static biological competition and compared biological and artificial visual sensitivities (Alam et al., 2014). Our work does not model the dynamics of bi-stable perception, it is only a window into the perceptual preferences and the image parameters predicting visual sensitivities, as well as the evolution of the inner preferences throughout the DNNs layers.

## 2. Methods

### 2.1. Data formation

**Figure 1:**
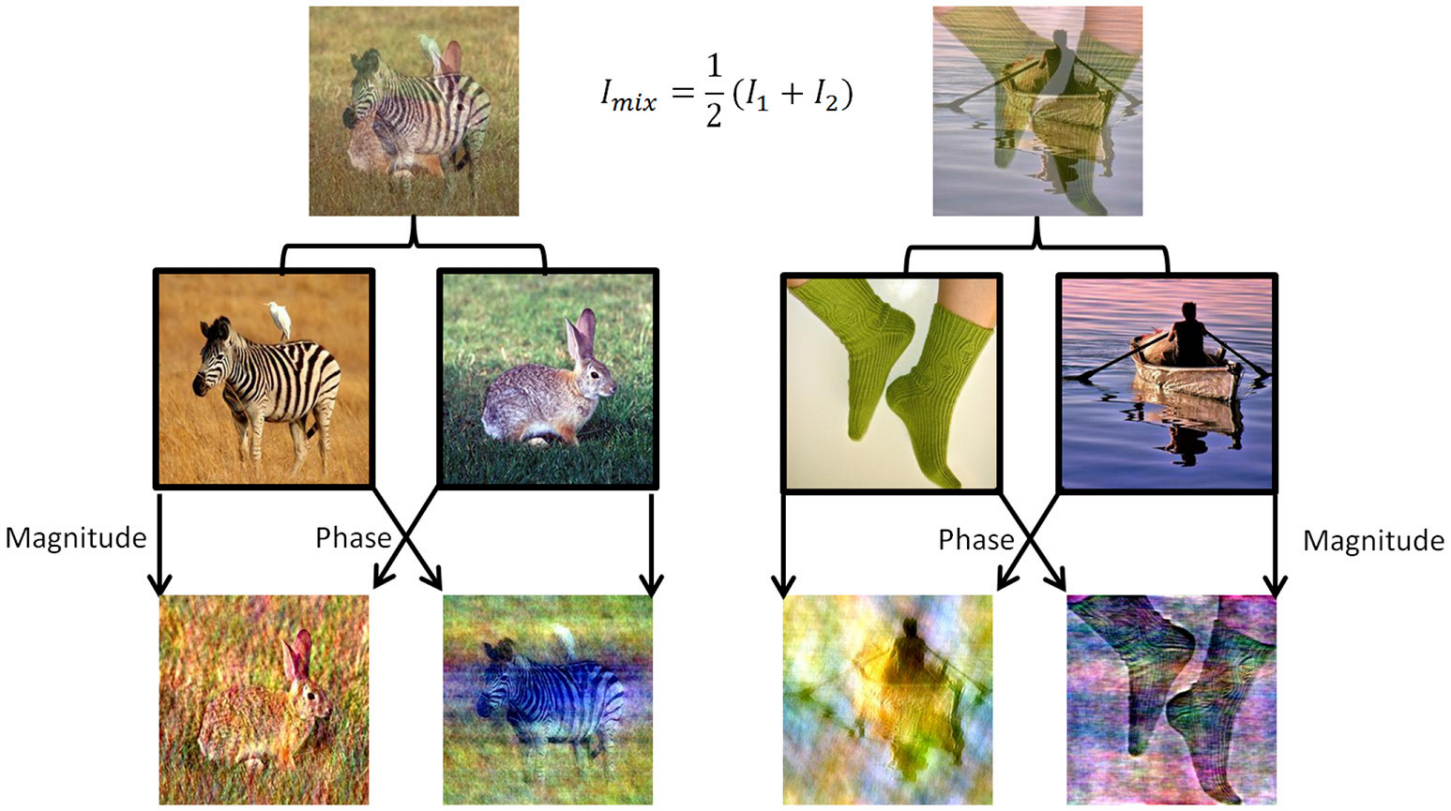
Two data sets of mixed images were created using images from the validation set of ImageNet. **(Top)** Example of the 50/50 morphing method (see text). **(Bottom)** Example of the phs/mag morphing method (see text). **(Middle)** Example of images from the original set.

To induce perceptual competition between two different visual stimuli that will enable us to test both human participants and DNNs algorithms we used ImageNet dataset (Russakovsky et al., 2015). We chose 180 images from different categories from ImageNet validation set and created mixtures of images in two morphing methods (Figure 1). For the DNN we generated all pairwise mixtures, and humans were tested on one set of unique mixtures. In the first method, named “50/50,” we averaged the RGB values of all pixels in the two images (Figure 1, top row). In the second method, named “phs/mag,” we Fourier-Transformed each image to get its magnitude and phase values in the frequency domain, then used the magnitude of one image with the phase from the other image, and transformed back using the inverse Fourier-Transform to get the final mix (Figure 1, bottom row). The second morphing method was inspired by a known visual phenomenon, according to which humans are sensitive to the phase rather than the magnitude of frequencies in natural images (Thomson et al., 2000).

### 2.2. DNN output classification

**Figure 2:**
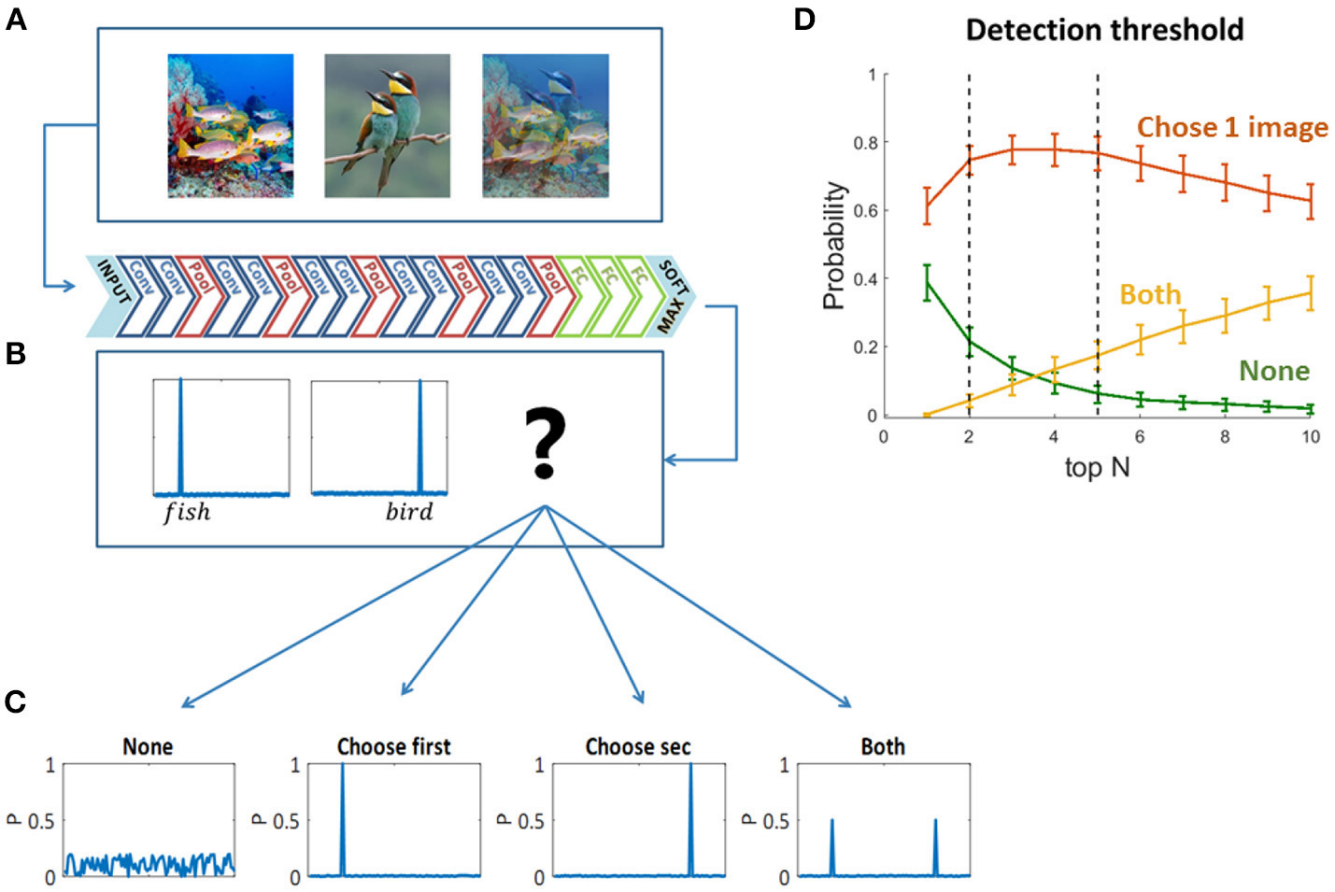
**(A)** One hundred and eighty original images and all pairwise mixtures between them were used as inputs to a pre-trained convolutional neural network (VGG19). **(B)** The network architecture. **(C)** Four possible softmax outputs when inserting a mixed image as an input (see text). **(D)** Detection threshold for output classification. The network choice was defined based on the overlapping top *N* categories of the original images and the mixed image (see text).

To decide which original image “wins” the visual competition, or which image is “chosen” by the network to be “perceived,” we used the two sets of mixed images as inputs to pre-trained feed forward convolutional neural networks (Figures 2A,B)—VGG19 (Krizhevsky et al., 2012; Simonyan and Zisserman, 2014) and ResNet (He et al., 2016). We chose VGG19 as a representative network based on its high performance in the ImageNet Large Scale Visual Recognition Challenge (ILSVRC). We preferred VGG19 over other similar networks due to its relatively high accuracy rate when tested on our dataset [Top5 accuracy: AlexNet-0.77, VGG S-0.83, VGG16-0.90, VGG19-0.92 (Krizhevsky et al., 2012; Simonyan and Zisserman, 2014)]. We also validated our results using ResNet (which achieved even higher accuracies than the above networks in ILSVRC, and a Top5 accuracy 0.92 on our dataset), but here we present the results of VGG19 as it is more similar in depth and architecture to the networks used in previous studies presenting the similarities to the primate ventral stream (Cadieu et al., 2014; Yamins et al., 2014; Kubilius et al., 2016; Yamins and Dicarlo, 2016). We then compared the output probability vectors of the SoftMax layer when the input was each one of the original images and when the input was their mix. We classified the output vectors of the mixed images to four types of scenarios (Figure 2C): the network did not choose any of the images; it chose the first image; the second image; or both of them. We defined “choosing an image” based on the top *N* categories in the output probability vectors: if one of the top *N* categories of the mixed image is also one of the top *N* categories of an original image—we say that the network chose to see this original image. In other words, we look for the top *N* categories of the mixed image in each of its two original images top *N* categories, if found—we consider that original image “chosen.” In this study we mainly used *N* = 5, as it is leading metric when testing classification DNNs with 1,000 categories, due to the use of over-specific categories in the data set. ImageNet is a single label dataset containing images that can fall into several categories and the order of those categories is ambiguous. Moreover, we show the network choices for *N* = 2 as well, which is the smallest relevant *N* for this task. We have verified that using a different *N* within this range did not change the preceding analysis, as the dominance of choosing one is highly similar for *N* = 2 and *N* = 5, and it does not change the winning image within each pair (red curve in Figure 2D). We randomly sampled 90 mixed images and calculated the probability of each scenario (none, choose one image, both). For each *N*, we averaged these probabilities over 100 iterations. To account for the stochastic nature of human choices (Moreno-Bote et al., 2007, 2011), we further calculated the network choices when injected with Gaussian noise in the last layer before the SoftMax. Hence, the output layer is given by:

(1) $$\begin{array}{l} P\left(class_{i}\right)=\frac{exp\left(x_{i}+noise\right)}{\sum_{i}exp\left(x_{i}+noise\right)},\;noise=N\left(0,\sigma^{2}\right) \end{array}$$

We again averaged over 100 iterations, with changing the standard deviation of the noise (σ) from 0 to 5. We present the level of noise that best resembled human choices. We have further verified that using the noise-injected results did not change the preceding analysis, similar to using top2 accuracy, as explained above.

### 2.3. Human experiment

The 180 images were uniquely paired to avoid repetitions that might cause memory biases. The 90 pairs were randomly divided to three groups of 30 mixtures each, yielding six conditions (three of the “50/50” and three of the “phs/mag,” github.com/lirongruber/Visual-Competition/tree/master/human%20experiment/img). We used Amazon Mechanical Turk to test 600 participants in an on-line experiment, 100 per condition (participants were 36.6 ± 10.6 years old, 303 of them were males). Ethics approval was obtained by the IRB (institutional review board) of the Weizmann institute of science. Each participant signed an informed consent form before participation and was paid 0.5$.

Each trial began with 1 second of fixation (+ at the screen center) followed by the brief image presentation. We presented the mixed images to participants for 100 ms (different browsers cause jitters of 7.5 ± 0.7 ms), as this brief exposure allows full recognition of regular images, while challenges the recognition of objects in the mixed images (Sheinberg and Logothetis, 1997; Cadieu et al., 2014). This time frame is commonly used in similar studies as it eliminates the effect of eye movements which enable humans to resample the image and impair the comparison (see Fig2S in Cadieu et al., 2014; Rajalingham et al., 2018).

Each trial ended with a free written report, usually between one to three words. Participants were instructed to report the object or objects they perceived, or type “none” if no object was recognized (empty reports were not accepted). Even though the networks rank 1,000 pre-determined categories, the open report is a better comparison than providing humans with a long list of options. An open report allows more authentic recognition answers, by not providing hints, not encouraging guessing and allowing the “none” option. Alternative solution as proposed in Kubilius et al. (2016) shortens the list but still has the above weaknesses of a closed report. Each written report was manually encoded to one of the four types of scenarios (Figure 2C). Decisions were made separately by two independent examiners, and the few disagreements were discarded (1.1%).

## 3. Results

### 3.1. Comparing DNN and humans choices

**Figure 3:**
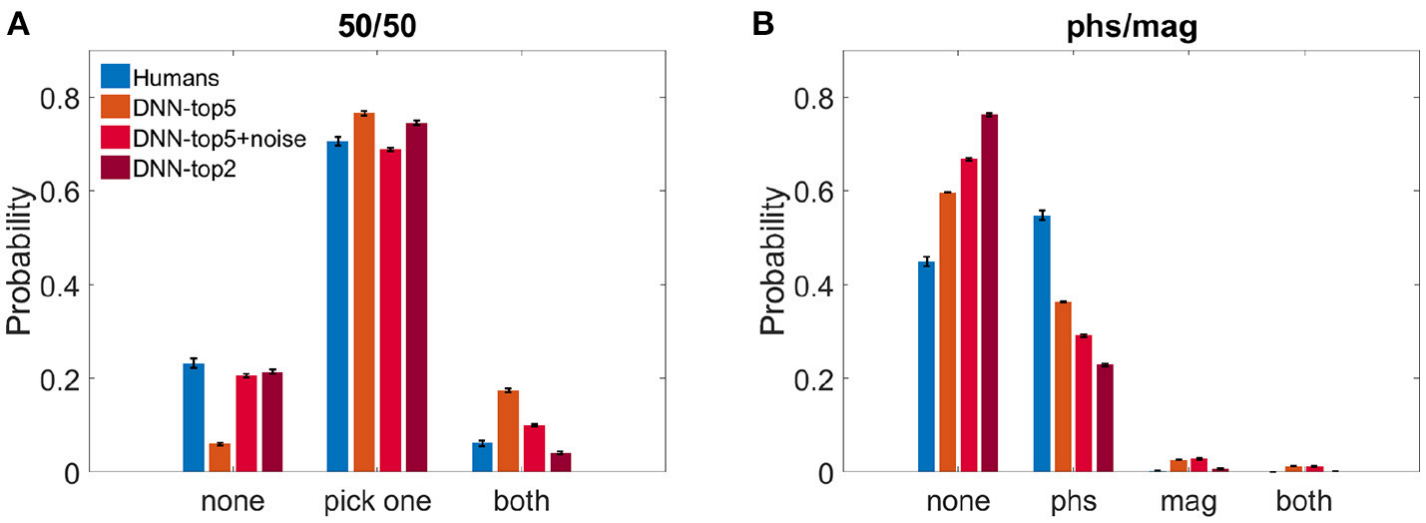
Histograms of choices classification. **(A)** DNN's top5, noise-injected-top5, top2 and humans' reports probability when observing the 50/50 dataset. **(B)** DNN's top5, noise-injected-top5, top2 and humans' reports probability when observing the phs/mag dataset.

We calculated the probability of both humans and the DNN to perceive either one image, both, or none of them. Figure 3A shows the results of the 50/50 dataset and Figure 3B shows the results of the phs/mag dataset, for VGG-19.

For the 50/50 case, humans reported recognizing only one image in 70.5 ± 1.6% of the trials. Similarly, the DNN chose only one image and suppressed the other in 76.5 ± 0.5% (ResNet—74.2 ± 0.4%) for *N* = 5 and 74.5 ± 0.4% for *N* = 2. For *N* = 5, the DNN successfully recognized the two images in 17.4 ± 0.4% (ResNet—18.5 ± 0.4%) of the trials and missed only 6.0 ± 0.3% (ResNet—7.1 ± 0.4%). On the other hand, humans recognized both images only in 6.0 ± 0.6% and reported not perceiving anything in 23.2 ± 1.7% of the trials. When using *N* = 2, the DNN successfully recognized the two images only in 4.1 ± 0.2% and missed 21.4 ± 0.4%. While this seems to better replicate the human results, one has to keep in mind the problematic use of the top2 accuracy rate, as described in the section 2. In an attempt to account for the stochastic nature of human choices compared with the deterministic one of the network, we injected Gaussian noise before the SoftMax layer of the network (see section 2).We present the DNN results with noise STD = 2.25, which best resembled human results: 20.6 ± 0.5% none, 68.8 ± 0.05% choose one image, 10.0 ± 0.3% both (Figure 3A).

On the other hand, in the phs/mag mixture, for *N* = 5, the DNN did not recognize any of the images in 59.6 ± 0.4% (ResNet -53.6 ± 0.4%) of the trials, while humans missed only 45.0 ± 1.0% of trials. In the recognized trials, humans always perceive the phase image (54.7 ± 1.0% of all trials) while the DNN is less sensitive to it (36.3 ± 0.4% of all trials, ResNet—42.1 ± 0.3%). While humans could never see the magnitude image, the DNN had a few successful trials of choosing it or both images (4.0 ± 0.1% of all trials, chance level is 2.0%, ResNet—3.5 ± 0.1%). Using top2 results or the noise-injected ones only further damaged the network success rates, increasing the number of unrecognized images(Figure 3B).

### 3.2. Single parameters predictability

Out of the mixtures that were perceived as one image (Figure 3A, middle bars), only in 79.0% of the trials the DNN and humans chose the same image (humans mode). To further characterize the differences between them, we extracted image parameters that may predict the DNN's and humans' tendency to prefer specific images over others. Based on vision research dealing with perceptual dominance (Logothetis, 1998; Blake and Logothetis, 2002; Tong et al., 2006; Blake and Tong, 2008), we extracted 12 initial features (average red, blue, and green component, colorfulness, luminance, saturation, global contrast, local contrast, horizontal and vertical gradient, 2D gradient, low frequencies, high frequencies) and then chose the least correlated among them (Table 1). We calculated the probability of an image to be chosen over another image, as a function of the ratio between their parameters. To quantify the predictability of each parameter we fitted the probability with a logistic regression model (as in Equation 2 for a single parameter *i*), where the model parameter (|β|) represents the degree of predictability. By knowing the value of a predictive parameter, one can estimate with high probability which image will be chosen.

**Table 1:** Image parameters.

**Parameter**	**Description**
Gradient	$\underset{pixels}{\sum }{\left(\nabla image\right)}^{2}$
Low frequencies	$\overset{i=0.25\cdot \left(maxfreq\right)}{\underset{i=0}{\sum }}|FFT\left(image\right)|$
Luminance	< 0.299*R* + 0.587*G* + 0.114*B* >_*pixels*_
Global contrast	*std*(0.299*R*+0.587*G*+0.114*B*)
Colorfulness	$\underset{pixels}{\sum }\left(I^{2}+Q^{2}\right)$ [YIQ coordinate system ]
Saturation	$<\frac{255\left(max\left(R,G,B\right)-min\left(R,G,B\right)\right)}{max\left(R,G,B\right)}{>}_{pixels}$

**Figure 4:**
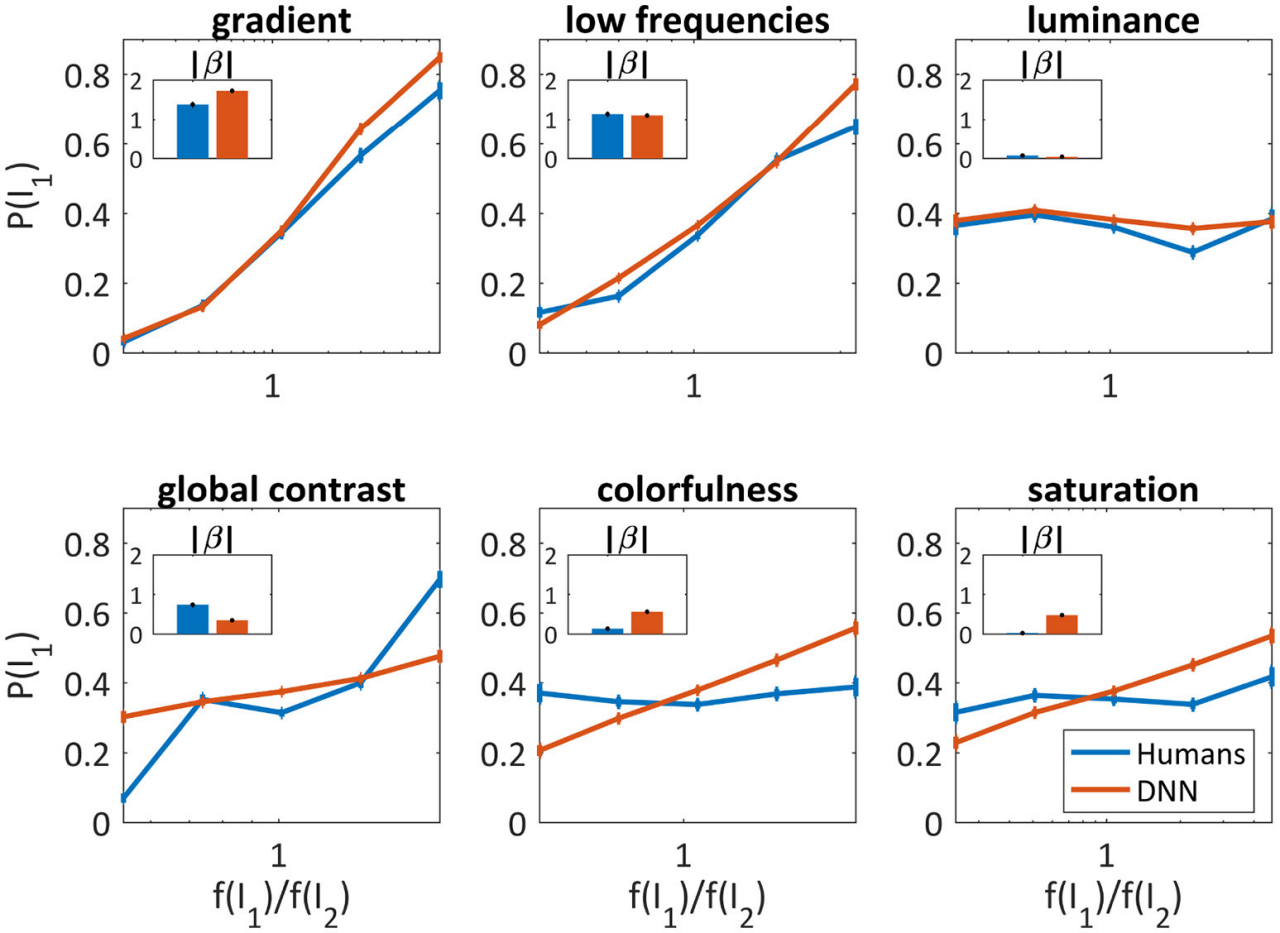
Image parameters as predictors for the DNN's and humans' choices for the 50/50 dataset (red and blue, respectively). The probability to choose *I*_1_ vs. $\frac{f\left(I_{1}\right)}{f\left(I_{2}\right)}$, where *f*(*I*_1_) is the parameter value of *I*_1_. X axis is log scaled. Error bars are the confidence intervals (95%) of a binomial distribution calculated with Clopper-Pearson method. Inner bar plots show β parameters of logistic regression (see text) for humans and DNN.

As can be seen in Figure 4, the gradient and the low frequencies were good predictors for both humans' (β = 1.38 ± 0.06, β = 1.14 ± 0.06, respectively) and the DNN's choices (β = 1.72 ± 0.05, β = 1.11 ± 0.04, respectively), and slightly better for the DNN in higher parameter ratios. The luminance was not at all predictive, again similarly for humans (β = 0.07 ± 0.04) and the DNN (β = 0.04 ± 0.03). Differences were found for global contrast which was a better predictor for humans (especially in low and high ratios, β = 0.73 ± 0.05) compared to the DNN (β = 0.34 ± 0.03), and colorfulness and saturation seem irrelevant for humans (β = 0.13 ± 0.04, β = 0.02 ± 0.04, respectively) while predicting to some extent the DNN's choices (β = 0.56 ± 0.03, β = 0.47 ± 0.03, respectively).

### 3.3. Multiple parameters predictability

We next looked for combinations of parameters that could increase the predictability. We optimized a regularized generalized linear model (GLM) for each subset of our six parameters and calculated the average prediction accuracy. The regularization parameter was determined via cross validation. As the two classes were balanced [P(pick *I*_1_) = P(pick *I*_2_)] we optimized a non-biased model (intercept = 0).

(2) $$\begin{array}{l} P\left(pick\;I_{1}|I_{1},I_{2}\right)=\frac{1}{1+exp\left(\sum_{i}\beta_{i}log\left(\frac{f_{i}\left(I_{1}\right)}{f_{i}\left(I_{2}\right)}\right)\right)} \end{array}$$

*I*_1_, *I*_2_ are the images, $\frac{f_{i}\left(I_{1}\right)}{f_{i}\left(I_{2}\right)}$ is the ratio of parameter *i* between the images, and β_*i*_ is the coefficient of parameter *i*. After the model was trained, the decision and accuracy were calculated using:

(3) $$\begin{array}{l} y^{model}=\left\{ \begin{array}{cc} 1,\; & P>0.5\\ 0,\; & P<0.5 \end{array}\right. \end{array}$$

(4) $$\begin{array}{l} accuracy=\frac{1}{N}\sum |y^{model}-y^{net}| \end{array}$$

*y*^*model*^ is the model choice (1/0 for choosing the first/second image, respectively), *y*^*net*^ is the DNN choice, and *N* is the number of images in each test set.

**Figure 5:**
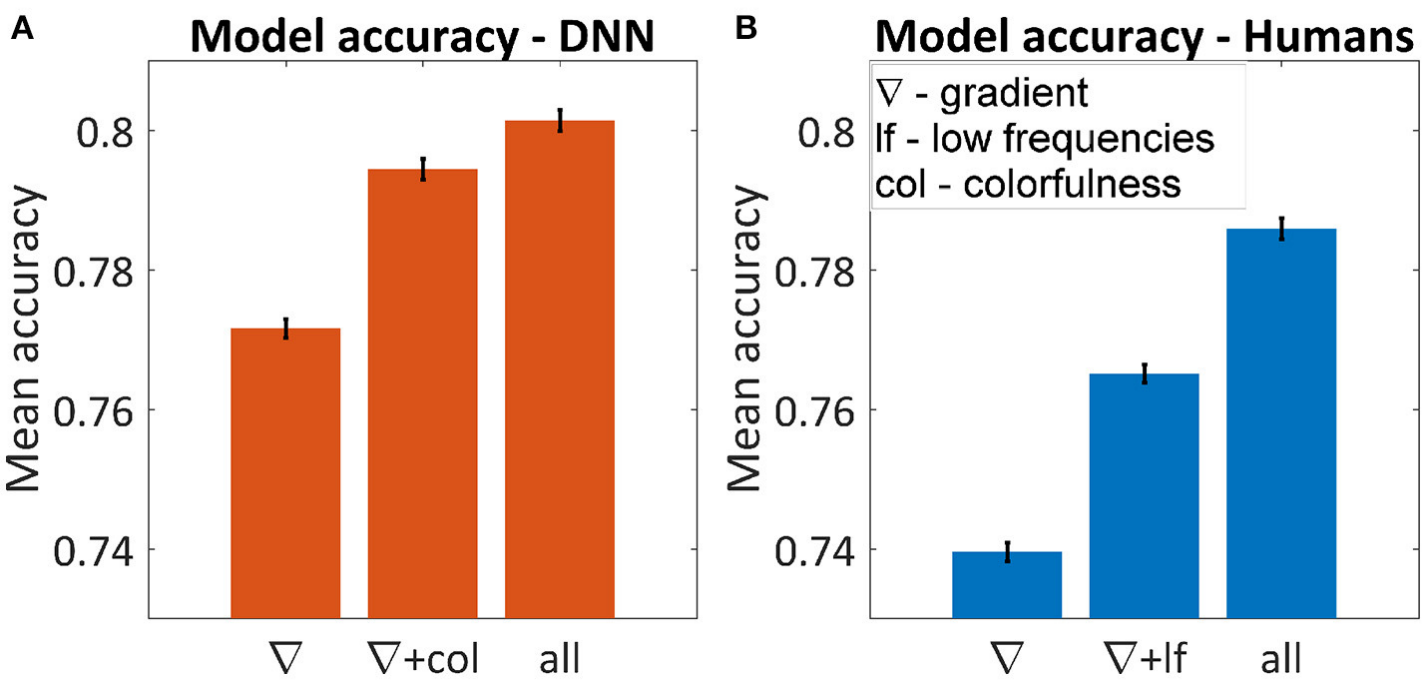
Average accuracy in predicting the winning image using multi-dimensional GLM for the DNN **(A)** and humans **(B)**. The figures represent the best subset of parameters when using one or two parameters and the maximum accuracy when using all of them. Using all six parameters yielded the same result as adding any third one in both cases. Error bars represent standard errors.

Figure 5A shows the average accuracy of the best subset for one, two and six parameters. The best single parameter for both humans and the DNN was the gradient, which predicted the DNN's and humans' choice in 77.2 and 74.0% of the cases, respectively. The best pair of parameters was different, for humans adding the low frequencies yielded 76.5% successes and for the DNN adding colorfulness reached 79.4%. The best accuracy achieved for the DNN was 81.0% and for humans 78.6%. In both cases, using all parameters was not significantly different than adding any third parameter.

### 3.4. Activity throughout the DNN layers

#### 3.4.1. 50/50 mixed images

**Figure 6:**
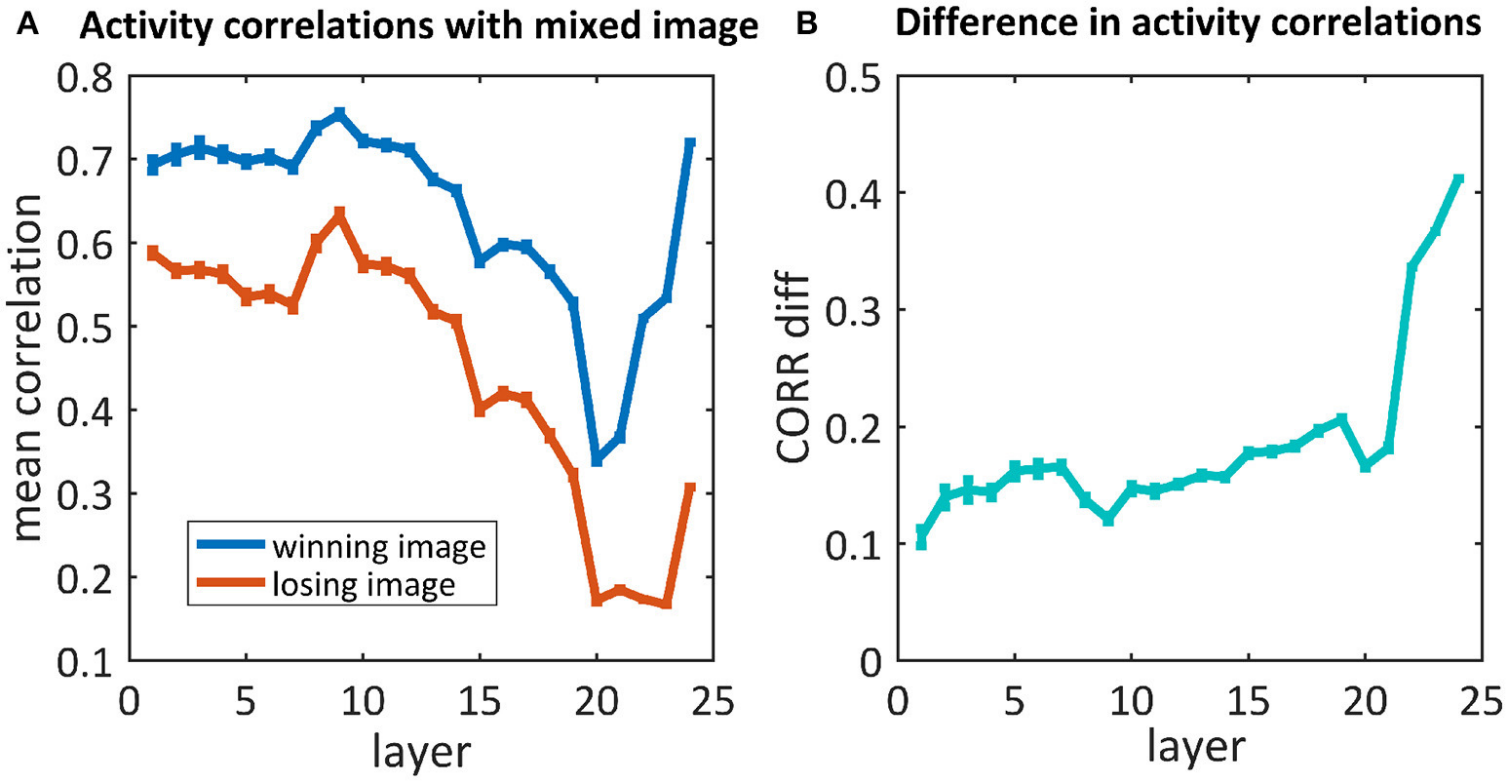
**(A)** The average correlations between the activity maps of the 50/50 mixed image units and the winning/losing image (blue/red) units. **(B)** Differences between correlations of the winning and losing image [i.e., difference between the blue and red curves in **(A)**, respectively].

As we are also interested in where this kind of competition is resolved, we further examined the activity of the network throughout the process of categorization, before the last softmax layer. We compared the activity of each neuron in each layer of the network when “observing” each of the original images and their mix. We calculated the correlations between those activity maps and averaged them per layer. To understand where the network's “decision” occurred, we calculated the average activity map correlations when averaging the “winning” images separately from the “losing” images (Figure 6). For both cases, the correlations in the first layers were high (0.7/0.6), decreased as we went deeper into the net and increased toward the end. When looking at the difference between these correlations (Figure 6B), although a difference already existed in the first layers, it increased dramatically in the last three layers. Suprisingly, we did not find any effect before/after max pooling (layers 3, 6, 11, 16, 21). On the other hand, the dramatic increase occurs in the fully connected layers (layers 22, 23, 24).

#### 3.4.2. phs/mag mixed images

**Figure 7:**
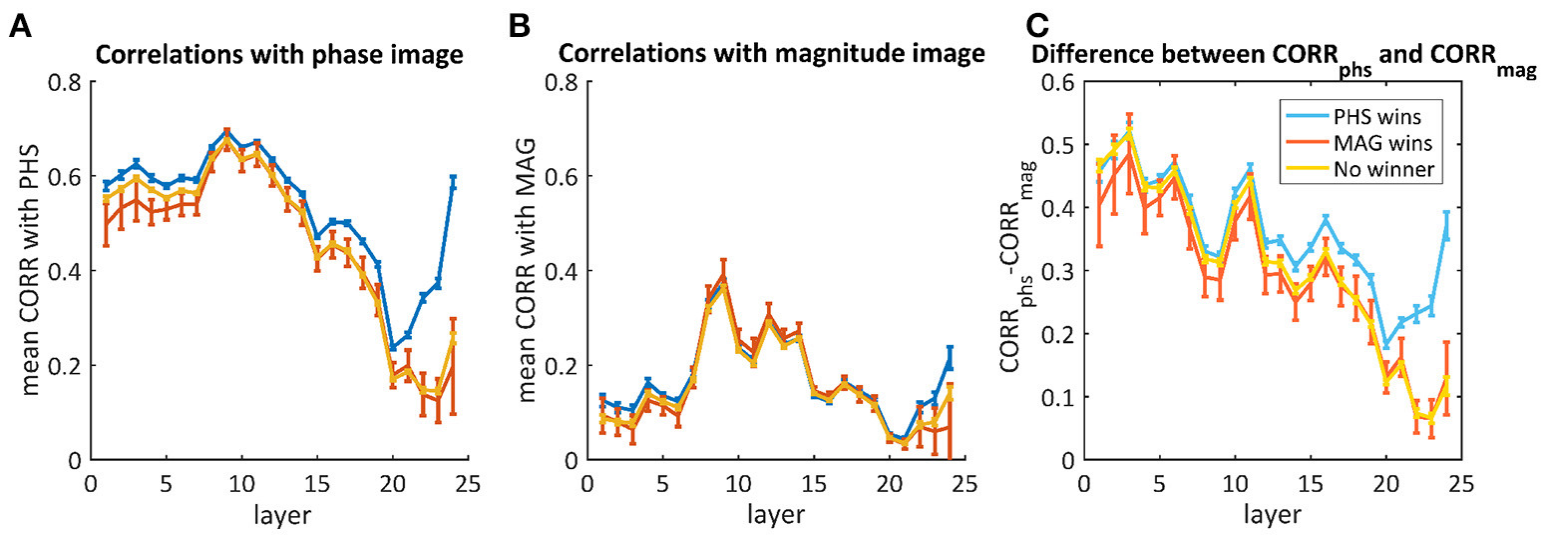
The average correlations between the activity maps of the phs/mag mixtures and the original phase image **(A)** or the magnitude image **(B)**. The difference between them is presented in **(C)**. The blue curves represent images where the network chose the phase image as the “winner,” the red is when the magnitude image “won” and the yellow is for the cases where neither “won.” Error bars represent standard errors.

Though most of the times the network did not recognize both images, we aim to understand whether there was a different response throughout the layers when it did recognize one of them. Therefore, we averaged separately the mixtures where the net chose the phase image, the magnitude image or neither. Figure 7 shows the average correlations throughout the layers with the phase image (Figure 7A), the magnitude image (Figure 7B), and the difference between them (Figure 7C). According to Figure 7C, there is a big difference in favor of the phase image already in the first layers, but this cannot be used as a predictor as it happened also for images where the magnitude image “won” (red) or neither (yellow). In the cases where the “phase” image “won,” the decision occurred only toward the end, where we observed a higher difference between the correlation with the phase image and the correlation with the magnitude image.

## 4. Discussion

To these days, a key challenge for neuroscientists is describing and understanding the nature of computation in the brain (Marr and Poggio, 1976). The rising success of artificial DNNs in object recognition tasks raises new questions about their resemblance to computations in the human visual system. Does the similarity between the biological and artificial systems goes beyond high accuracy? This study asserts a connection between deep networks and the human visual processing mechanism, adding to a growing body of studies showing that DNNs can be used for modeling different phenomena of the visual system (Cadieu et al., 2014; Khaligh et al., 2014; Yamins et al., 2014; Güçlü and van Gerven, 2015; Kubilius et al., 2016; Cichy et al., 2017; Seeliger et al., 2017). It further reveals still existing divergence for future model improving. In this study, we have created a non-dynamic human visual competition. When briefly presented with a mixture of two images humans tended to perceive only one image (70.7%). Remarkably, when testing DNNs on the same mixes, only one of the images appeared in the top5 categories of the DNN (VGG19—76.3%, ResNet—74.2%). Using the top5 categories is the leading evaluation metric for networks with 1,000 categories, and specifically when working with the ImageNet dataset. The categories of this dataset are over-specific as they contain types of animals and parts of objects (e.g., green mamba, Passerina cyanea, modem, nail, etc.). Some of the images may also fall into more than one category (e.g., the man on the boat from Figure 1). As our goal was to determine which of the images was better perceived, or better popped-up in the brief exposure, we accepted any human answer referring to any part of an image, as well as used the top5 categories of the network. Moreover, we have verified that evaluating the network perception by choosing top2 categories would not change the main tendency to perceive only one image. This result implicates that the “suppression” of the unperceived stimulus can be explained without any top-down processes, using only a feed-forward architecture. While referring to the network's output as perception is still controversial, we refer here to a narrower definition which is the task related categorization. Our visual task involves two stimuli competing for the system's perception—whether biological or artificial. This comparison is powerful, as the exact same stimulus was presented to both humans and a DNN.

While using only the top2 categories seemed to cover-up the discrepancies in perceiving both images or none of them, we believe, for the reasons listed above, it is a worse candidate for comparison to humans. Although, when using top5 accuracy, one has to account for a discrepancy in performance. In the current dataset and using the top5 categories, the net recognized both images at almost three times the rate of humans (Figure 3A). One plausible source for this difference is the deterministic nature of the DNN, compared with the stochastic one of humans. Inspired by studies using noise to model human stochasticity (Daw et al., 2006; Moreno-Bote et al., 2007, 2011), we examined the effect of injecting noise to the decision-making process of the network. We showed that adding noise before the last layer enabled reaching similar to human results. In other words, the disparities we have mentioned so far might result from the lack of stochasticity in the DNN. Important to mention, though, is that neither using top2 accuracy nor noise-injection changed the winning image within each pair. Hence, it strengthens the robustness of the tendency to perceive only one image, and cannot account for all following similarities and differences found in the preceding analysis.

Finally, we note that humans were better than the DNN at recognizing images in the phase/magnitude mixtures (Figure 3B), and that this advantage was mainly due to increased sensitivity to the image phase. This sensitivity was previously shown to reflect natural images variability (Thomson et al., 2000), and our finding implies that the DNN model we used is lacking in this regard.

We further attempted to regress performance of both systems to image attributes. Our analysis revealed that frequencies, both high (as captured by the gradient) and low, are common predictors of humans' and the DNN's choices. The influence of image gradient on human perception had been previously shown in different paradigms (Hollins, 1980; Mueller and Blake, 1989), here, we show that this sensitivity exists also for the DNN model. On the other hand, although commonly used in psychophysical studies, the luminance was not a good predictor for either the DNN or for humans. Global contrast was a good predictor only for human performance, which might be explained by the low resolution enforced by the short exposure, while colorfulness and saturation were predictive only for the DNN's choices. The DNN's sensitivity to colorfulness was also observed using a generalized linear model, which further emphasizes the gradient's role as the common and most predictive parameter.

The parameters which predicted performance similarly for both systems may now offer a platform on which computational explanations to human sensitivities may be tested. These visual sensitivities spontaneously emerge from training an artificial system for classification, suggesting a similar mechanism in biological systems. Parameters which predicted performance differently point to a possible disparity between the two perceptual implementations—the biological and the artificial. These differences may aid vision researchers in developing more human-like artificial networks, e.g., reducing network's sensitivity to color by augmenting the training dataset with color manipulations. Alternatively, one can re-train the networks using the mixed images labeled with human's choices.

Finally, we attempted to resolve where in the computational process perceptual competition was resolved. The activity throughout the layers of the DNN indicates that a preference for the perceived image existed already in early processing levels, though the difference in the last layers increased dramatically. This late preference in the fully-connected layers was also observed in the phase/magnitude competition. This result is consistent with a previous study, showing that in neural networks trained for binary choices, information regarding both choices can be tracked throughout the layers (Balasubramani et al., 2018). It is further consistent with the primary functions of the different layers, convolutional layers serve as feature extractors, while fully-connected layers are in charge for the classification (Hertel et al., 2015).

Our results offer a two-fold benefit for future work. First, they can be used to improve the validity of DNNs as models, as well as boost their performance (by imitating biological behaviors). Second, testing DNNs outputs using manipulated inputs provide a new approach for vision researchers to study how the brain makes choices of what to perceive. In conclusion, this work is yet another step toward a valid computational model of the ventral stream of the visual system. The differences we found can be used for bridging the gaps between biological and artificial visual perception.

## Data availability statement

The dataset generated for the human experiment and the results can be found in https://github.com/lirongruber/Visual-Competition.

## Author contributions

LG and AH designed the research, conducted the human experiment, analyzed the data and wrote the paper. RB and MI supervised the analysis and contributed by reviewing and editing the manuscript.

### Conflict of interest statement

The authors declare that the research was conducted in the absence of any commercial or financial relationships that could be construed as a potential conflict of interest.
